# Brain–Computer Interface: The HOL–SSA Decomposition and Two-Phase Classification on the HGD EEG Data

**DOI:** 10.3390/diagnostics13172852

**Published:** 2023-09-03

**Authors:** Mary Judith Antony, Baghavathi Priya Sankaralingam, Shakir Khan, Abrar Almjally, Nouf Abdullah Almujally, Rakesh Kumar Mahendran

**Affiliations:** 1 Department of Computer Science & Engineering, Panimalar College of Engineering, Chennai 600123, India; 2 Department of Computer Science & Engineering, Amrita School of Computing, Amrita Vishwa Vidyapeetham, Chennai 601103, India; 3 College of Computer and Information Sciences, Imam Mohammad Ibn Saud Islamic University (IMSIU), Riyadh 11432, Saudi Arabia; sgkhan@imamu.edu.sa (S.K.); aamjally@imamu.edu.sa (A.A.); 4 University Centre for Research and Development, Department of Computer Science and Engineering, Chandigarh University, Mohali 140413, India; 5 Department of Information Systems, College of Computer and Information Sciences, Princess Nourah bint Abdulrahman University, Riyadh 11671, Saudi Arabia; 6 Department of Computer Science and Engineering, Rajalakshmi Engineering College, Chennai 602105, India; rakeshkumarmahendran@gmail.com

**Keywords:** brain–computer interface (BCI), electroencephalogram (EEG) signals, artifact removal, Singular Spectrum Analysis (SSA), Independent Component Analysis (ICA)

## Abstract

An efficient processing approach is essential for increasing identification accuracy since the electroencephalogram (EEG) signals produced by the Brain–Computer Interface (BCI) apparatus are nonlinear, nonstationary, and time-varying. The interpretation of scalp EEG recordings can be hampered by nonbrain contributions to electroencephalographic (EEG) signals, referred to as artifacts. Common disturbances in the capture of EEG signals include electrooculogram (EOG), electrocardiogram (ECG), electromyogram (EMG) and other artifacts, which have a significant impact on the extraction of meaningful information. This study suggests integrating the Singular Spectrum Analysis (SSA) and Independent Component Analysis (ICA) methods to preprocess the EEG data. The key objective of our research was to employ Higher-Order Linear-Moment-based SSA (HOL–SSA) to decompose EEG signals into multivariate components, followed by extracting source signals using Online Recursive ICA (ORICA). This approach effectively improves artifact rejection. Experimental results using the motor imagery High-Gamma Dataset validate our method’s ability to identify and remove artifacts such as EOG, ECG, and EMG from EEG data, while preserving essential brain activity.

## 1. Introduction

EEG is a technique for detecting electrical activity in the brain. Since the electrodes are often positioned along the scalp, it is noninvasive. EEG readings that are aberrant are the outcome of the most frequent use of the technology, which is to diagnose epilepsy according to [1]. Additionally, it may be utilized to spot brain death, coma, encephalopathies, sleep disorders, and the degree of anesthesia. For identifying tumors, strokes, and other focal brain illnesses, among other conditions, EEG was previously considered the gold standard. The use of this technology has decreased, nevertheless, owing to the advancement of good structural imaging methods such as computerized tomography and magnetic resonance. EEG continues to be a vital study and diagnostic tool despite its poor spatial resolution [2]. CT, PET, and MRI cannot really compete with its millisecond-range temporal resolution. EEG is often tolerant of subject mobility, unlike the majority of other neuroimaging methods. To achieve a better analysis of the reactions to auditory stimuli, it is also possible to reduce motion abnormalities in EEG data.

Before analyzing EEG data, unnecessary components must be eliminated, which is highly essential to achieve improved accuracy. Therefore, source separation approaches in EEG signal processing have gained a great deal of attention. Mixed data have independent sources that are statistically concealed. For source separation, the Blind Signal Extraction (BSE) and Blind Source Separation (BSS) approaches were suggested [3]. Independent Component Analysis (ICA) and Principal Component Analysis (PCA) were more often employed in many studies to separate the signals that are concealed in the mixed EEG data [4]. The most popular method for effectively separating the sources from the EEG signal’s complex requirements is called ICA, and it belongs to the BSS class. The PCA approach has certain limitations, such as a poor ability to convert directions. When it comes to separating sources and artifacts from EEG data, the ICA approach is more precise and versatile [5]. Because they utilize less power, wearable and portable EEG devices have become more popular. With these gadgets, it is simple to monitor and record EEG signals at home. Additionally, there is spectral overlap between the EEG and the source components.

To suppress the artifacts from multichannel EEG recordings, Independent Component Analysis has been primarily used to address these differences. The use of ICA in real time is not possible with systems that have only one or a few EEG channels. Using ICA to eliminate artifacts on a single channel is substantially more difficult. The suggested work emphasizes separation using ICA. The challenging job is in building a technique that can separate artifacts from a single-channel EEG signal. The direct use of single-channel EEG signals with ICA cannot be done. Whereas, for multichannel EEG signal processing, ICA techniques are used more often. Therefore, the single-channel signal is mapped into multivariate data using the appropriate decomposition technique for overcoming the limitation. The single-channel signal was mapped into multivariate data using Wavelet Transform and EEMD, respectively, by the authors in [6]. However, this technique failed to separate the sources efficiently according to the report.

A decomposition method that is frequently applied in the study of meteorological time series data is Singular Spectrum Analysis (SSA) [7]. A tensor-based SSA method was utilized to extract the narrow band variable using the Empirical Mode Decomposition (EMD). However, this requires many calculations during the SSA reconstruction step. In SSA, a Finite Impulse Response filter was later described using a truncated SVD, with the eigenvectors serving as the filter coefficients. Recently, SSA has been utilized to create filter banks. In such implementations, the output remained in phase with the original signal.

The primary advantage of the suggested strategy over the existing SSA–ICA and SSA–ANC [8] procedures is the capacity to separate the sources present in single-channel EEG signals in a multiview data analysis. In other words, the suggested technique of decomposition translates the signal into multivariate data with many dimensions, as opposed to existing multivariate data that only have spatial and temporal dimensions, which is referred to as Higher-Order SVD-based Singular Spectrum Analysis as a consequence. The recommended HOL–SSA technique is utilized to deconstruct the single-channel signals into multivariate data, which are then subsequently used to recover the source signals using the Online Recursive ICA (ORICA) approach.

This study introduces an innovative methodology for identifying signal sources within single-channel EEG data. The approach involves combining the Singular Spectrum Analysis and Independent Component Analysis techniques. Specifically, we propose the utilization of a novel method called Higher-Order L-moment Singular Value Decomposition-based SSA (HOL–SSA). This technique is a linear combination of Higher-Order Singular Value Decomposition (HOSVD), as demonstrated by [9]. HOL–SSA has exhibited superior robustness compared to both higher- and lower-order statistical methods within SSA.

The proposed manuscript is organized as follows. Section 2 explains the proposed approaches of HOL–SSA and its algorithm. Section 3 explains the dataset used for experimentation, the results obtained, and their analysis. Finally, Section 4 concludes the entire work and its benefit with direction toward future enhancement.

## 2. Literature Survey

The authors in [10] introduced a new method based on the Singular Spectrum Analysis (SSA) technique for classifying brain activity based on EEG signals via an application into a benchmark dataset for epileptic study. The results from the SSA-based approach were compared with those from discrete wavelet transform. It finds that SSA can capture both stationary and nonstationary EEG features more effectively than wavelet transforms. The automated removal of EOG artifacts from EEG signals was presented by authors in [11]. Circulant Singular Spectrum Analysis (CiSSA) was employed by them to decompose the EOG-contaminated EEG signals into intrinsic mode functions (IMFs). Subsequently, the artifact signal components were identified through the utilization of kurtosis and energy values, and their removal was executed by means of a four-level discrete wavelet transform (DWT). The results indicate that the proposed approach was evaluated on synthetic and real EEG data, revealing its effectiveness in the elimination of EOG artifacts while retaining low-frequency EEG information.

In their study [12], the authors introduced a novel and effective technique for the removal of muscle artifacts from EEG signals. The method, named SSA–CCA (Singular Spectrum Analysis–Canonical Correlation Analysis), combines Singular Spectrum Analysis (SSA) and Canonical Correlation Analysis (CCA). Unlike conventional single-channel decomposition methods, such as ensemble empirical mode decomposition (EEMD), the SSA algorithm employed in this approach draws on principles of multivariate statistics. This enables the proposed method to harness the benefits of both SSA and cross-channel information. The efficacy of SSA–CCA is assessed using both semi-simulated and real EEG data. The results of the evaluation reveal that the introduced method surpasses existing techniques, namely, EEMD–CCA, and even the classic approach of CCA, particularly when dealing with multichannel scenarios. This innovative SSA–CCA approach thus presents a promising advancement in the domain of EEG artifact removal.

As the successful elimination of EOG artifacts remains a significant obstacle in EEG research, the authors proposed a novel approach, termed EEMD-based ICA (EICA) [13]. This method combines ensemble empirical mode decomposition (EEMD) with ICA algorithms to enhance the removal of EOG artifacts from multichannel EEG signals. However, when conducting a comparative analysis, the authors found that the Singular Spectrum Analysis (SSA) method exhibits superior performance. SSA showcases the highest improvement in signal-to-noise ratio, coupled with a reduction in root mean square error and correlation coefficient after the removal of EOG artifacts. This robust performance of SSA underscores its ability to more effectively eliminate blink artifacts from multichannel EEG signals, while minimizing the impact of error. As a result, SSA emerges as a promising solution for addressing the challenge of EOG artifact removal in the realm of multichannel EEG signal analysis.

One emerging approach that has gained attention in recent years is the two-phase classification approach, which involves a sequential classification process aimed at enhancing accuracy, efficiency, and noise reduction. This review highlights the merits of the two-phase classification approach in comparison to other classification methods commonly used in EEG signal processing. 

The seminal work by the authors in [14] discusses the conceptual framework and practical implementation of a two-stage classification approach as compared to single-stage classifiers. By leveraging multiple stages, the proposed methodology enables the model to first capture high-level patterns and subsequently refine predictions in the second stage. Empirical evidence presented in this article underscores the improved accuracy, generalization, and adaptability of the two-stage classifier across diverse datasets.

In the comparative study, the authors systematically assess the performance of single-stage classifiers against a two-stage classifier using multiple datasets [15]. The article meticulously outlines the benefits of the two-stage approach, which includes superior feature extraction and hierarchical decision-making. The experimental results clearly illustrate that the two-stage classifier consistently outperforms single-stage alternatives, emphasizing the efficacy of its intricate decision pipeline.

Focusing on the complexities posed by intricate datasets, the article [16] by the authors elucidates the merits of employing a two-stage classification strategy. Through an in-depth examination of real-world scenarios, the authors demonstrate the limitations of single-stage classifiers and how the two-stage approach is better suited to handle such challenges. By effectively segmenting the decision-making process, the proposed methodology showcases remarkable performance improvements, establishing its relevance in intricate data analysis.

The authors in [17] have presented a case study that highlights the tangible benefits of adopting a two-stage classification model in practical applications. Drawing from a specific domain, they outline the shortcomings of using single-stage classifiers and present evidence of the two-stage model’s remarkable success. Similarly, the authors in [18] introduced a dual-stage classification approach. In the initial stage, they employed LDA classifiers to distinguish between various pair-wise MI tasks. Following this, a naive Bayes classifier was employed to forecast the ultimate task executed by the user. This prediction is based on the weighted results of the LDA classifiers. The conducted experiments indicated that the proposed method surpassed the top-performing entry in BCI competition IV by a margin of 3.5%.

Through careful analysis and extensive experimentation, this work underscores the superiority of the two-stage classification approach, reinforcing its viability in real-world scenarios.

The proposed study contributes an adaptive two-phase classification technique for MI events, showcasing improved accuracy and consistency in BCI performance. The study by the authors in [19] presents a method for epileptic seizure detection in EEG signals, leveraging nonlinear features and a deep learning model. Both studies highlight the significance of innovative classification methodologies in distinct domains, with the first emphasizing enhanced performance in BCI and the second demonstrating exceptional accuracy in epileptic seizure detection using advanced feature extraction and DL techniques.

The proposed study in this research and the study in [20] addresses classification challenges in distinct domains utilizing advanced methodologies. In Study 1, the emphasis is on MI event classification using a two-phase approach, with ANN and adaptive SVM classifiers. The adaptive technique aims to improve BCI performance by maintaining consistency, reducing training time, and handling non-stationarities. Study 2, on the other hand, focuses on epileptic seizure detection in EEG signals, employing a comprehensive CADS. It incorporates TQWT decomposition, extraction of various features, and a CNN-RNN DL model for classification. Both studies demonstrate significant improvements over existing approaches. Moreover, the proposed model can be efficiently used for other applications of medical images segmentation for brain data studies.

## 3. Methods and Materials

### 3.1. Singular Spectrum Analysis (SSA)

Singular Spectrum Analysis (SSA) is a powerful technique to handle time series data [21]. It can handle nonlinear and nonstationary time series data. SSA has shown great promise in the analysis of electroencephalography (EEG) signals [22]. It is a data-driven technique which identifies the alpha, beta, gamma, etc., associated with different brain activities. The processing steps of SSA include: (1) Embedding, (2) Singular Value Decomposition, (3) Grouping, and (4) Reconstruction.

The proposed contribution is HOL–SVD-based decomposition in the SSA rather than the conventional SVD. HOL–SSA is a linear combination of Higher-Order Singular Value Decomposition (HOSVD). It proved to be more robust than the existing higher-order and lower-order statistics of SSA. Both HOSVD and SVD are matrix factorization techniques, they handle higher or multidimensional data. HOSVD can handle nonlinear data. It can handle complete spatial and temporal features from EEG data simultaneously, making it useful for analyzing data with complex spatiotemporal patterns. SVD does not capture the full spatiotemporal patterns in EEG data. HOSVD can handle missing data in the tensor by using tensor completion, whereas SVD requires a complete matrix for analysis. However, the choice of method will depend on the specific application and the characteristics of the data being analyzed.

### 3.2. HOL–SSA

Multiple approaches to SSA were proposed for decomposition. Here, it is proposed to use a novel Higher-Order L-moment Singular Value Decomposition-based SSA (HOL–SSA), a linear combination of Higher-Order Singular Value Decomposition (HOSVD), which has been proved to be more robust than the existing higher-order and lower-order statistics of SSA. The recommended HOL–SSA technique is utilized to deconstruct the single-channel signals into multivariate data, which are then subsequently used to recover the source signals using the Online Recursive ICA (ORICA) approach.

#### 3.2.1. HOSVD

Most frequently, the multidimensional SVD is associated with the extraction of relevant information from the multiway cluster. A Multilinear Singular Value Decomposition is another term that is used. The relevant data are sampled in several dimensions using the multidimensional digital signal processing technique. The process of performing single-dimensional samplings involves selecting points along a continuous line and recording their values in a data stream. Contrarily, in multidimensional sampling, the data are chosen using a matrix based on the dataset’s sample vectors. The Tucker compression, which is a method for reducing the amount of multidimensional data, is mostly implemented using the HOSVD.

For tensor *R* of order *O* and size $s_{1}x{\;s}_{2\;}x$……x${\;s}_{O\;}$, the HOSVD is defined as follows.

(1) $$R=C_{R}\;x_{1}\;P^{\left(1\right)}\;x_{2}\;P^{\left(2\right)}\;x_{3\ldots \ldots \ldots }x_{O}\;P^{\left(O\right)}$$

where $C_{R}$ is the core tensor.

$P^{\left(m\right)}$ are the matrices of m-mode singular vectors of *R* with m=1,2,….O.

For every m=1,2,….O, the following are determined.

- SVD of m-mode unfolding $R_{\left(m\right)}$ of *R* as $R_{\left(m\right)}=P^{\left(m\right)}.\;\Sigma^{\left(m\right)}.\;Q^{{\left(m\right)}^{T}}$
- After the computation of matrices of *m* mode singular vectors $P^{\left(m\right)}$, the core tensor $C_{R}$ can be computed as follows.
  (2) $$C_{R}=R\;x_{1}\;P^{{\left(1\right)}^{T}}\;x_{2}\;P^{{\left(2\right)}^{T}}\;x_{3\ldots \ldots \ldots }x_{O}\;P^{{\left(O\right)}^{T}}$$
- Number of nonzero diagonal elements in $\Sigma^{\left(m\right)}$ as the m rank.

Similar to *O* matrix SVDs in difficulty, the HOSVD of an order *O* tensor *R* is also computationally complex.

#### 3.2.2. Truncated HOSVD

An efficient and approximative solution is to compute the greatest m-mode singular values. After determining the dominant m-mode singular vectors’ matrices, derived from $R_{\left(m\right)}^{\prime }=P^{\left(m\right)}.\;\Sigma^{\left(m\right)}.\;Q^{{\left(m\right)}^{T}}$, the truncated core tensor $C_{R}^{T}$ is obtained from the elements of the core tensor $C_{R}^{\prime }$.

(3) $$R^{\prime }=C_{R}\;x_{1}\;P^{\left(1\right)}\;x_{2}\;P^{\left(2\right)}\;x_{3\ldots \ldots \ldots }x_{O}\;P^{\left(O\right)}$$

(4) $$C_{R}^{\prime }=R^{\prime }\;x_{1}\;P^{{\left(1\right)}^{T}}\;x_{2}\;P^{{\left(2\right)}^{T}}\;x_{3\ldots \ldots \ldots }x_{O}\;P^{{\left(O\right)}^{T}}$$

The term “truncated HOSVD” refers to the low *m* rank approximation *R’* of the tensor *R*, which has the dominating *m* ranks [23]. In several applications across a wide range of signal processing domains, the HOSVD has been employed. It is extremely promising to use the reduced HOSVD as a preprocessing step for several multilinear signal processing methods’ dimensionality reduction. Thus, the computational complexity may be greatly decreased.

#### 3.2.3. L-Moment

The L-moment analysis is a statistical method used to analyze the probability distributions. As the HOSVD method decomposes the EEG signal into its spatial, spectral, and temporal components, the L-moment provides the distribution information of each component. The approach provides support in identifying patterns in the signal that would not be apparent using traditional signal processing techniques thus providing more accurate and reliable results [24].

In statistical theory, using cumulants and joint cumulants for univariate and multivariate distributions is one well-established method for Higher-Order Statistics. These are extended in time series analysis to higher-order spectra, such as the bispectrum and trispectrum.

L-moments, which are linear statistics (linear combinations of order statistics) and thus more reliable than HOS, can be used as an alternative to HOS and higher moments. L-moments are a series of statistics that are used to condense a probability distribution’s form. The L-scale, L-skewness, and L-kurtosis are linear combinations of order statistics (L-statistics) that are comparable to traditional moments and may be used to derive numbers similar to standard deviation, skewness, and kurtosis, respectively, where the L-mean is identical to the conventional mean. Standardized moments are equivalent to standardized L-moments, also known as L-moment ratios. A theoretical distribution has a collection of population L-moments, similar to conventional moments. For a sample taken from the population, sample L-moments are established and utilized as estimators of population L-moments.

The *n*th population L-moment for random variable *Z* is

(5) n−1∑i=0n−1(−1)in−1i∗E∗Zn−i:n

where *E* stands for expected value and *Z_i:N_* represents the kth order statistic (*n*th least value) in an independent sample of size *N* from the distribution of *Z*.

The recommended HOL–SSA technique is utilized to deconstruct the single-channel signals into multivariate data, which are then subsequently used to recover the source signals using the Online Recursive ICA (ORICA) [25] approach (Algorithm 1).
**Algorithm 1:** λ2 = (EX2:2 − EX1:2)/2λ3 = (EX3:3 − 2EX2:3+EX1:3)/3Proposed HOL–SSA And ORICA Methodology**Step 1**:  **Input** the raw EEG signal.
**Step 2**:  **Map** the signal vector to a matrix. In the embedding stage, the time series s with length l is mapped into tensor $R^{\prime }$, where s is segmented using a nonoverlapping window of size i and a[l/i] x i matrix M is obtained from s. $$M=\left[\begin{array}{ccc} s_{1} & s_{2}... & s_{i}\\ s_{\begin{array}{c} i+1\\ .\\ .\\ .\\ .\\ . \end{array}} & s_{i+2}... & s_{\begin{array}{c} 2i\\ .\\ .\\ .\\ .\\ . \end{array}}\\ s_{\left(L-1\right)i} & s_{\left(L-1\right)i+1}\cdots & s_{Li} \end{array}\right]$$
L = [l/i]
  *L* refers to the last slab of the tensor. The matrix M is converted to tensor $R^{\prime }$ by considering each slab of the tensor as a windowed version of M.
Because the application of SSA to real data does not exploit the inherent nonstationarity and therefore may fail in actual data decomposition, therefore, tensor-based SSA is a robust solution to this problem.
**Step 3**:  **Decompose** the signal using HOSVD.
  The truncated HOSVD of the converted tensor $R^{\prime }$ of order O and the dominant m ranks for m=1,2,….O is computed.
**for** m=1,2,….O
{
**compute** $R_{\left(m\right)}^{\prime }=P^{\left(m\right)}.\Sigma^{\left(m\right)}.Q^{{\left(m\right)}^{T}}$
**compute** matrices of dominant m-mode singular vectors ${[P}_{1}^{\left(m\right)},P_{2}^{\left(m\right)},..,P_{dom}^{\left(m\right)}]$
}
**compute** $C_{R}^{\prime }=R^{\prime }x_{1}P^{{\left(1\right)}^{T}}x_{2}P^{{\left(2\right)}^{T}}x_{3\ldots \ldots \ldots }x_{O}P^{{\left(O\right)}^{T}}$

**compute** $C_{R}^{t}$ from $C_{R}^{\prime }$
**Step 4**:  **Determine** the Linear moments of HOSVD.

  The nth population L-moment of a tensor with O order statistics in a decomposed sample from the distribution of core tensor $C_{R}^{t}$ is as follows.=n−1∑O=0n−1(−1)O.n−1OE.CR(n−O:n)′
  E is the expected value.
**Step 5**:  **Reconstruct** the original signal to a multivariate data matrix.
The matrices from step 4 are grouped into submatrices, as given below.
$$\sum_{z=1}^{Y}M_{z}$$
Here, Y represents the total number of groups, z refers to the subgroups of eigenvalues, and $M_{z}$ denotes the sum of matrices within group z.
Secondly, each matrix of the grouped decomposition is Hankelized, after which the Hankel matrix is transformed into a new series of length $l^{\prime }$. The diagonal averaging applied to the resultant matrix produces a reconstructed series. Thus, the initial series set $s_{1},\ldots .,s_{l}$ is decomposed into a sum of r reconstructed subseries, as shown below.
$$s=\sum_{1}^{r}s^{1}$$
This decomposition is the main result of the HOL–SSA algorithm. If each reconstructed subseries is categorized as a single periodic component or noise, the decomposition makes sense. As a result, the online recursive ICA technique is used in this situation for component separation, as indicated in the step that follows.
**Step 6**:  **Apply** ORICA on the multivariate data matrix, and for each iteration, the whitening matrix and the demixing matrix are computed.
In order to reverse the mixing action, the inverse matrix of the reconstructed subseries is built. The independent components are produced by applying the ORICA rule after applying the Sherman–Morrison matrix inversion method.$S_{i+1}^{-1}=S_{i}^{-1}+l_{r}\left[I-f\left(a_{i}\right)*a_{i}^{T}\right]*S_{i}^{-1}$
$S^{-1}$ refers to the demixing matrix of the r reconstructed subseries.
**Step 7**:  **Output** the mapped sources of interest into original signal form. 
**Time Complexity:** $O{(N}^{3})+O(M)$


The characteristics of the denoised EEG data are then extracted using the Common Spatial Pattern (CSP) technique. A two-phase classification strategy has also been suggested and tested on the motor 4 imagery EEG data, which is likewise in accordance with this. Cross-comparison tests also demonstrated that the suggested two-phase classification approach including Artificial Neural Network and Adaptive Support Vector Machine has greater classification accuracy than the existing single-stage and two-stage classification approaches [26].

## 4. Result and Analysis

### 4.1. Dataset Description 

The suggested model is assessed using the HGD, a different dataset, to confirm its resilience to data fluctuations. The HGD contains four classes—left hand, right hand, both feet, and rest—and more trials than the BCI-IV 2a. Fourteen individuals provided the HGD, which was gathered in a controlled environment. Just 21 of the 128 channels used to acquire the data, which had a sampling frequency of 500 Hz, were associated to MI.

The HGD dataset’s data quality was improved by downsampling it from 500 Hz to 250 Hz. In addition, channels were reduced from 128 to 21 in order to discard redundant information. Electrodes that do not link to the motor imagery region are left out. As the database description states, only 21 sensors with the letter C in their name were chosen since they represent the motor cortex. 

### 4.2. Performance Analysis

**Figure 1:**
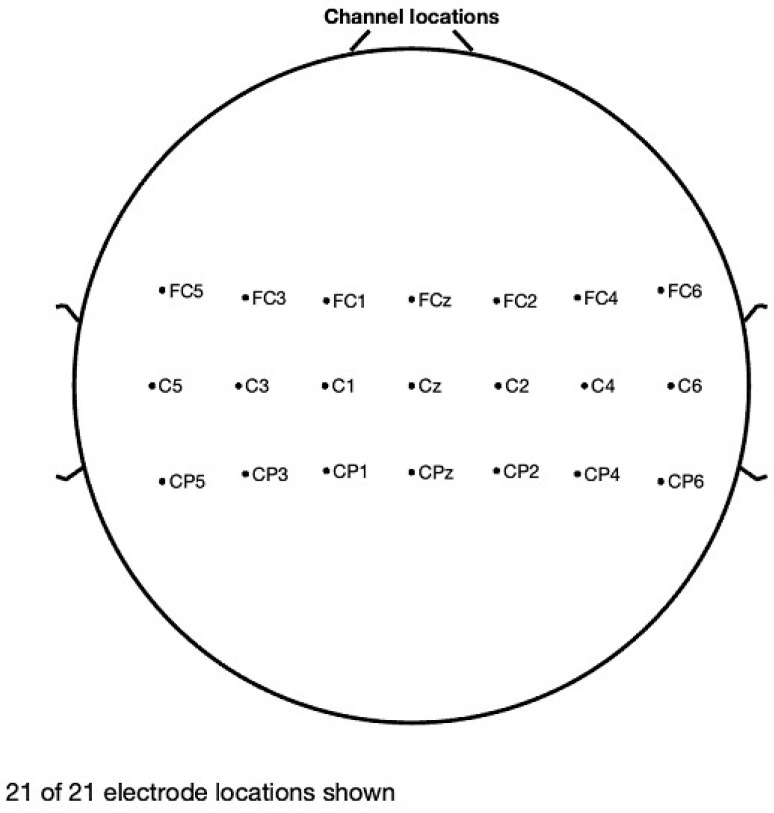
Channel Locations.

**Figure 2:**
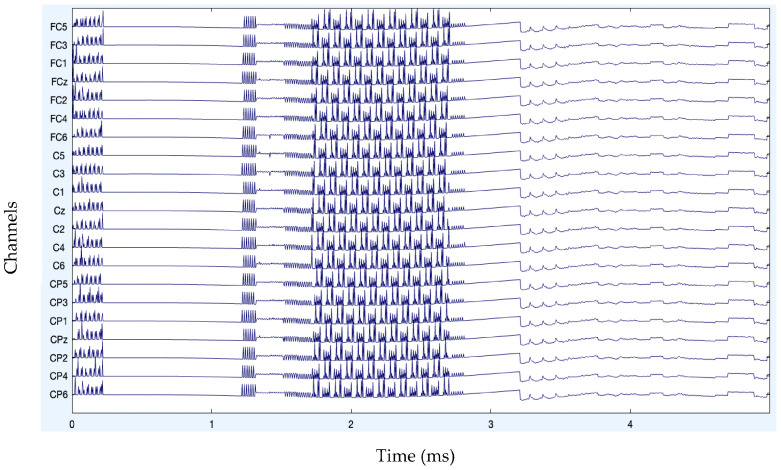
Original channel data of MI signals.

The analysis of artifact removal on the HGD motor imagery signals using the proposed approach is discussed below. Figure 1 represents the channels used to acquire the motor imagery signals and their locations. The signals acquired by each of these channels are represented in Figure 2 as channel data. These signals are further decomposed using the proposed decomposition approach. 

Note that executing ICA requires that bad channels be rejected first. The entire dataset should be cycled through in order to visually detect faulty channels because some of them could only be harmful intermittently. In this instance, removing the erroneous data segment rather than the channel itself may be better. Plotting the channels’ spectra is another approach to spot problematic channels. Bad channels might be rejected using the pop select.m function if they are known. Moreover, as filtering might scatter the artifacts out over clean data, necessitating additional data to be discarded after filtering, it may be desirable to remove data parts containing substantial artifacts by visual examination, such as high spikes in the data, before filtering.

**Figure 3:**
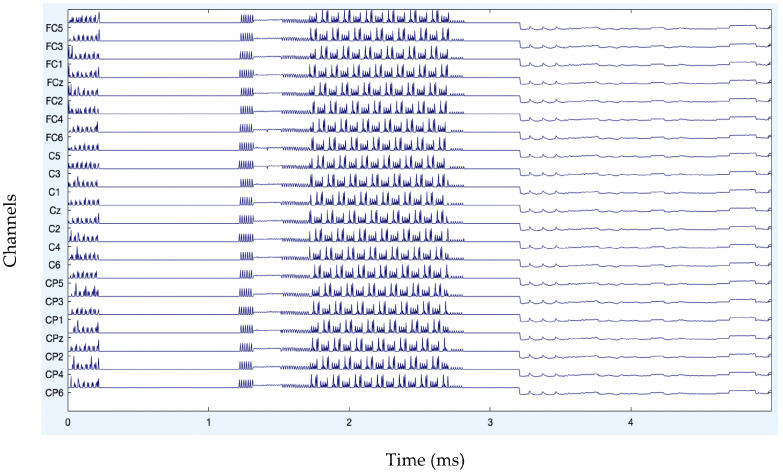
Channel data after filtering.

**Figure 4:**
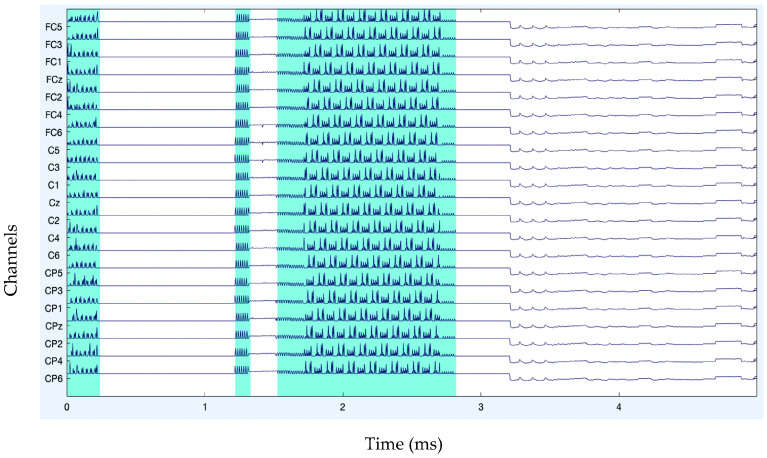
Rejection of bad data.

**Figure 5:**
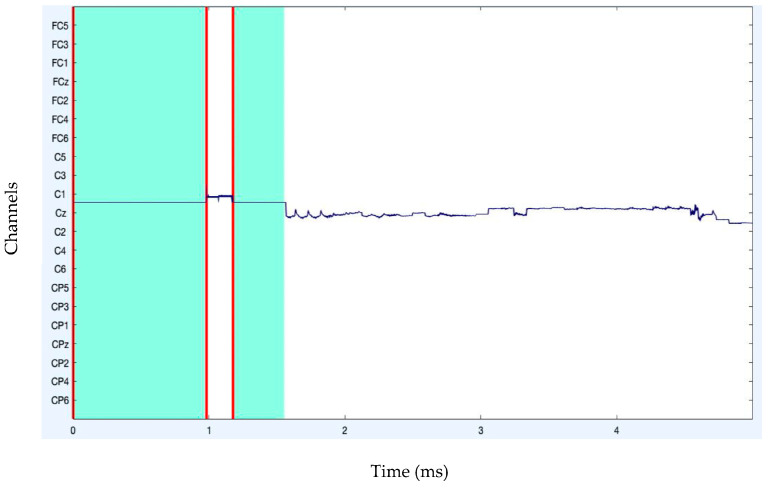
Rejection of bad data (stacked form).

After band-pass filtering of the signals, Figure 3 shows the channel data. Before filtering, it is also preferable to eliminate data segments having significant artifacts by visual inspection, such as large spikes in the data. Problematic data segment deletion is seen in Figure 4 and Figure 5. 

Although epoched data can also be filtered, screening continuous EEG data before epoching or artifact removal is advised since it reduces the introduction of filtering artifacts at epoch borders. It may be beneficial to high-pass filter the data to eliminate linear trends. It is recommended to apply high-pass filtering to the data at 1 Hz to generate signal decompositions of high quality.

**Figure 6:**
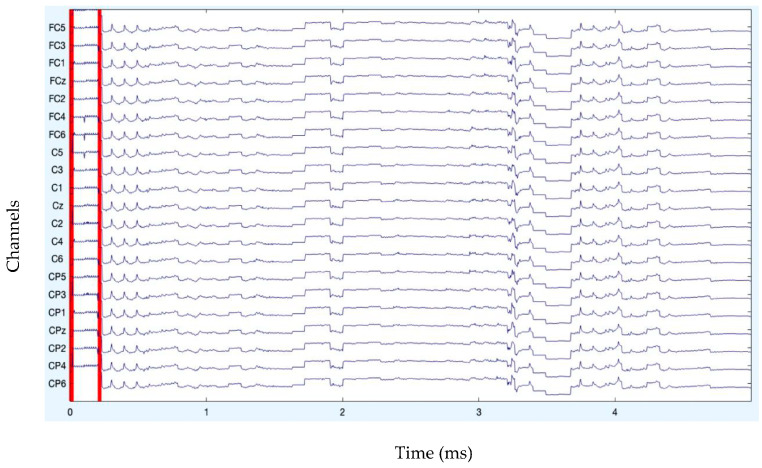
Boundary creation after data rejection.

Moreover, when large artifacts are removed, as seen in Figure 6, a “border” event replaces the deleted data. It is possible to reject or remove any portion of the continuous data in the eegplot.m box. After portions of the data have been flagged for rejection, a new dataset will be created. Any part of the continuous data in the eegplot.m box could be rejected or deleted. A new dataset will be constructed when some sections of the data have been designated for rejection.

The components are listed in decreasing order of the EEG variation that each component accounts for. EEG datasets always contain eye artifacts. They frequently occupy the top spots in both their scalp topographies and component arrays.

**Figure 7:**
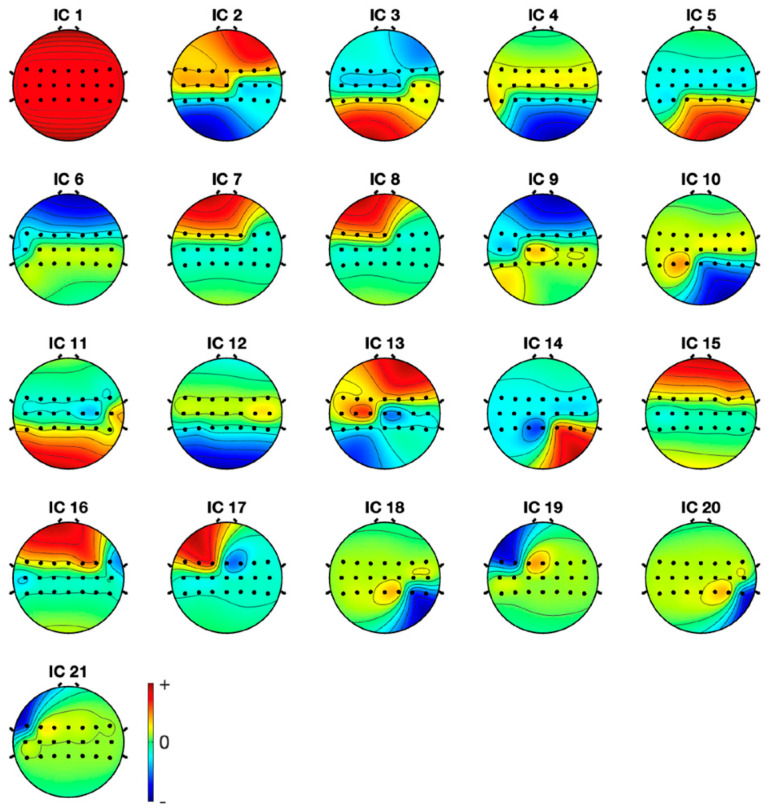
Topoplots of the independent components.

**Figure 8:**
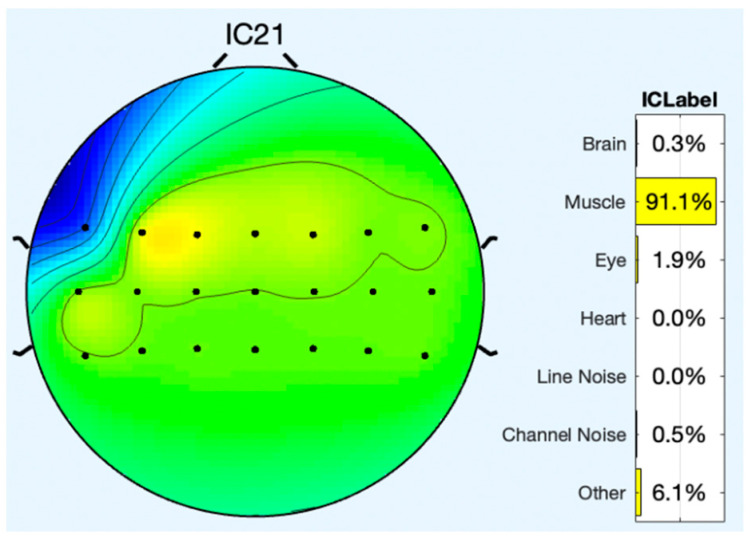
Scalp map of component 21.

**Figure 9:**
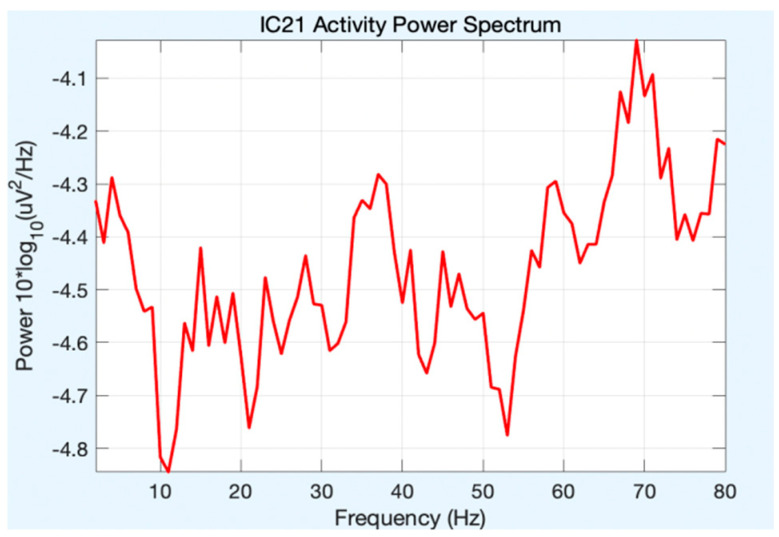
Activity power spectrum of component 21.

**Figure 10:**
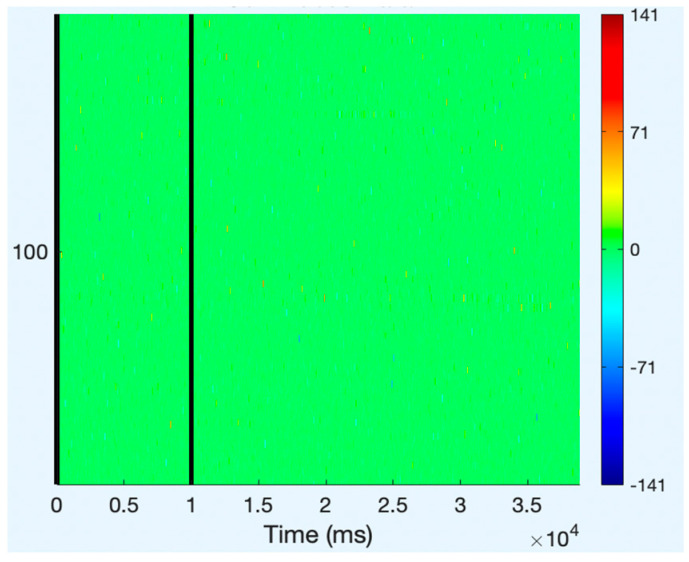
ERP of component 21.

All of the component topoplots are shown in Figure 7. Figure 8’s depiction of the scalp map for component 21 illustrates the existence and volume of artifacts in the EEG data. This component appears to have a significant level of muscular artifacts, and Figure 9 displays the corresponding activity spectrum. Ocular artifacts, which typically occupy the highest locations in their scalp topographies, can be seen together with EEG data. As a consequence, component 21 may be identified as an eye artifact since neither the findings of the ERP in Figure 10 nor the scalp map shows a significant far-frontal projection that characterizes eye artifacts.

**Figure 11:**
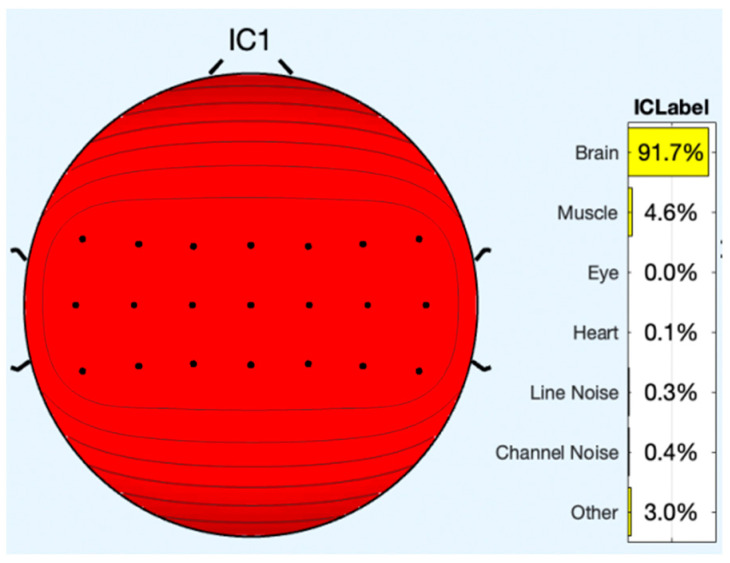
Scalp map of component 1.

**Figure 12:**
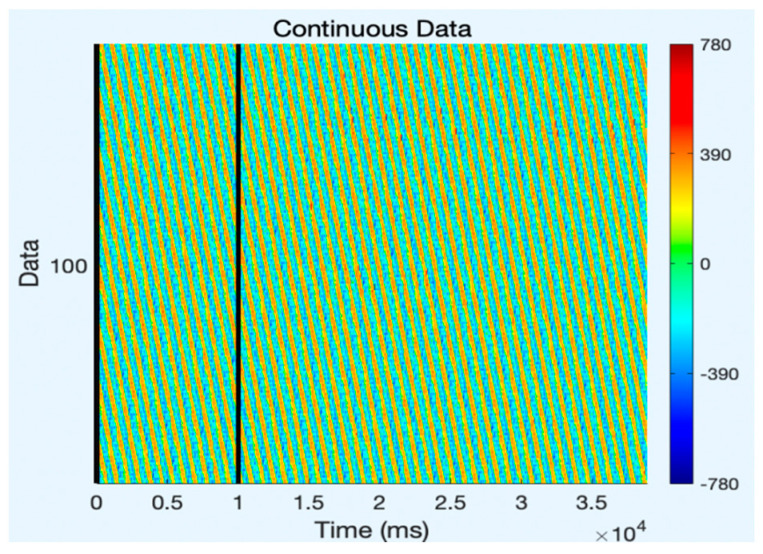
ERP of component 1.

**Figure 13:**
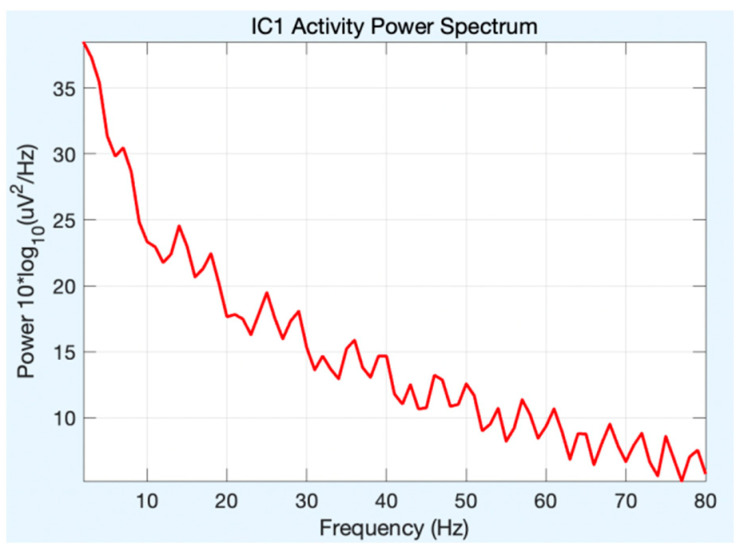
Activity power spectrum of component 1.

Relatively, Figure 11 depicts the scalp map of component 1, which has fewer artifacts and more EEG signals. Figure 12 and Figure 13, respectively, display the activity power spectrum and ERP map of the same. Table 1 lists the artifacts that are present in each component.

**Figure 14:**
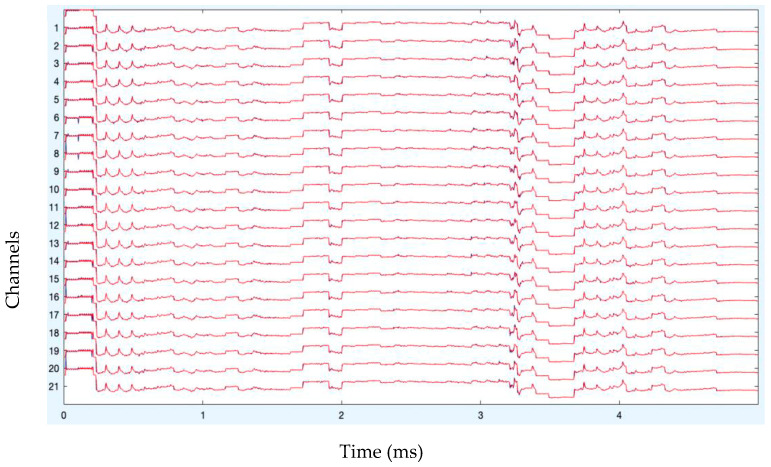
Pruned data after artifact removal.

**Figure 15:**
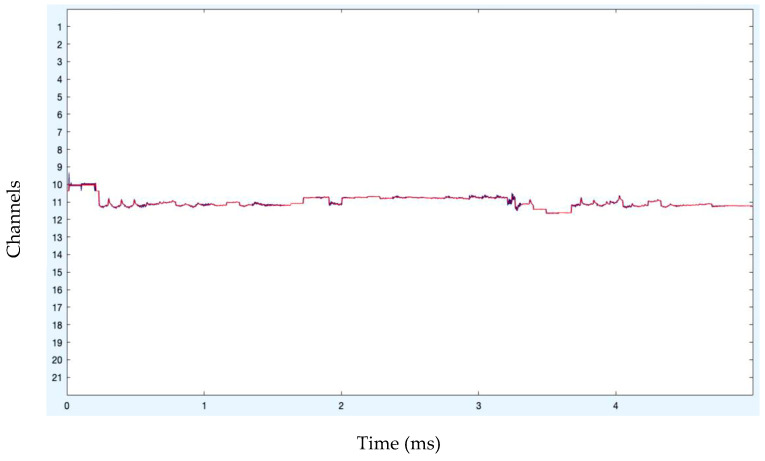
Pruned data after artifact removal (stacked form).

Following artifact removal, the pruned data are eventually shown in Figure 14 and Figure 15, where the artifact-free signals are depicted in red. So, it is found to be quite advantageous to remove artifact regions that include unique artifacts while generating pure independent components. The signals that have had the artifacts removed are then transmitted for feature extraction and classification. The following chart compares the classification performance of the artifact-free HGD motor imagery signals using the proposed ANN + A-SVM model to other approaches tested on the identical HGD motor imagery EEG signals. The classification performance is evaluated under different metrics such as accuracy, precision, recall, K-score, F1-score, and misclassification rate. The accuracy reported is 95.24%, with an average K value of 0.94. Also, the precision, recall, F1-score evaluated for the four classes (Left, Right, Feet and Rest) of all the 14 subjects are reported in the analysis, as shown in Table 2. 

**Figure 16:**
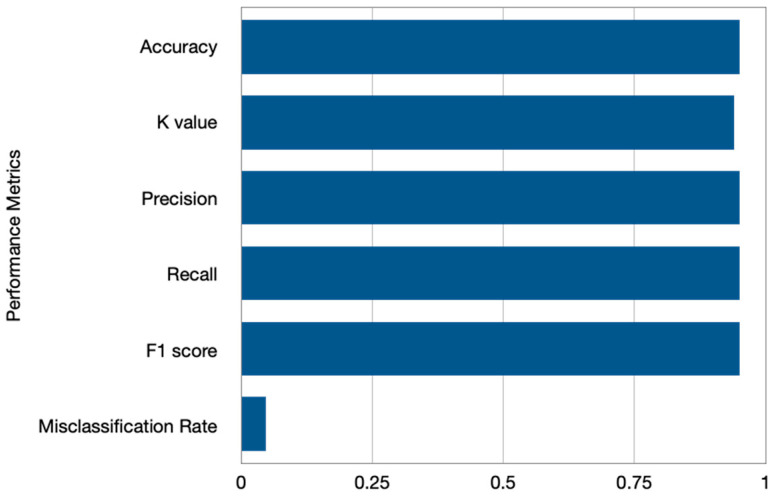
Classification performance under Accuracy, K-Score, Precision, Recall, and F1-Score metrics on HGD dataset.

**Figure 17:**
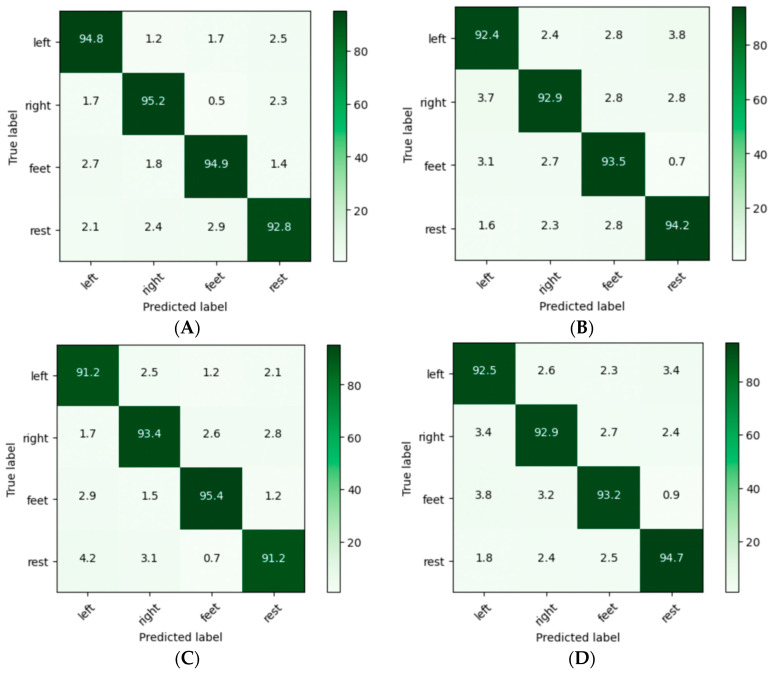
Confusion matrices of subjects (**A**) S4, (**B)** S5, (**C**) S13, and (**D**) S14.

**Figure 18:**
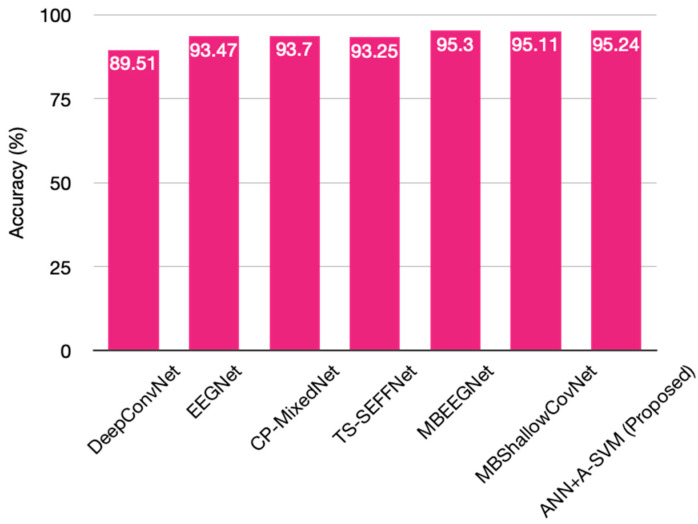
Comparison of classification performance chart.

The average misclassification rate of 0.047 is better compared to the existing approaches. This performance analysis is graphically represented in Figure 16. The classification performance is also represented through the confusion matrices in Figure 17. The confusion matrices are shown for four subjects, S4, S5, S13, and S14, where the prediction values are found to be better. Table 3 shows the performance comparison between the proposed models and other models. In particular, the classification accuracy of every subject and the average classification accuracies obtained by the DeepConvNet, EEGNet, CP-MixedNet, TS-SEFFNet, MBEEGNet, and MBShallowCovNet from the HGD dataset is summarized in Table 3. Our method has the highest average accuracy of 95.24%, except for the MBEEGNet approach, which has an accuracy of 95.30%. The comparison is graphically presented in Figure 18.

Table 4 shows the performance comparison between the proposed models and other models. The average classification accuracies from the BCI-IV 2a and HGD Motor Imagery datasets are summarized in the table. Using the two public datasets, the performance of the proposed model is evaluated where it has proved to perform better compared to the other models.

## 5. Conclusions

In this research article, a new method for removing artifacts from EEG signals has been put forward. The proposed HOL–SSA involves a Higher-Order Linear-Moment-based approach to decompose the signal into multivariate data followed by the ORICA method to separate the sources. The suggested HOL–SSA and ORICA approach performs better compared to a number of other current decomposition and source separation approaches. Thus, the proposed HOL–SSA and ORICA-based preprocessing approach has shown improved results in artifact rejection. The experimental findings demonstrate that the suggested technique can identify and eliminate EOG, ECG, EMG, and other artifacts from EEG data while still preserving brain activity that is ignored by the noise component. The computational complexity of the suggested artifact removal approach is also shown in the algorithm. The ANN + A-SVM, two-stage classifier improves the classification performance on the HGD motor imagery dataset, as shown in Table 3 and Table 4.

## Figures and Tables

**Table 1:** EEG and artifacts present in the observed signals.

Components/Signals	EEG (%)	Muscle (%)	Eye (%)	Heart (%)	Line Noise (%)	Channel Noise (%)	Other (%)
**IC 1**	97.1	4.6	0.0	0.1	0.3	0.4	3.0
**IC 2**	10.0	85.2	0.6	0.0	1.4	0.1	2.6
**IC 3**	3.3	93.3	1.0	0.0	0.8	0.0	1.6
**IC 4**	1.6	95.9	0.6	0.0	0.6	0.0	1.2
**IC 5**	2.1	95.2	0.7	0.0	0.6	0.0	1.3
**IC 6**	0.9	84.7	9.0	0.0	0.3	0.0	5.0
**IC 7**	0.6	66.7	22.4	0.0	1.2	0.1	9.0
**IC 8**	0.2	73.0	5.3	0.0	0.4	0.3	20.9
**IC 9**	0.9	93.5	0.4	0.0	0.8	0.1	4.3
**IC 10**	6.8	50.6	1.3	0.4	8.2	0.3	32.2
**IC 11**	7.6	83.7	1.2	0.3	1.2	0.1	5.9
**IC 12**	12.7	77.4	0.2	3.3	1.2	0.1	5.2
**IC 13**	14.0	40.1	0.9	0.2	5.3	3.2	36.4
**IC 14**	12.3	63.0	0.7	1.1	3.5	0.1	19.2
**IC 15**	1.0	37.9	13.2	0.0	0.3	0.4	47.2
**IC 16**	1.3	90.1	1.9	0.0	0.8	0.3	5.6
**IC 17**	2.4	60.2	3.1	0.1	0.2	0.6	33.5
**IC 18**	5.7	47.7	2.8	0.5	1.5	0.6	44.2
**IC 19**	1.9	67.7	1.1	0.0	0.4	0.8	28.1
**IC 20**	0.7	82.2	0.9	0.2	0.3	0.8	15.1
**IC 21**	0.3	91.1	1.9	0.0	0.0	0.5	6.1

**Table 2:** Classification performance on the HGD dataset using the proposed model.

Participants		S1	S2	S3	S4	S5	S6	S7	S8	S9	S10	S11	S12	S13	S14	Avg.
**Accuracy (%)**		94.2	96.4	94.4	95.6	93.9	96.2	96.7	94.2	94.9	95.2	96.9	96.5	94.6	93.7	**95.2**
**K value**		0.92	0.95	0.93	0.94	0.92	0.95	0.95	0.92	0.93	0.94	0.96	0.95	0.93	0.92	**0.94**
**Precision**	**LH**	0.93	0.97	0.93	0.96	0.93	0.97	0.96	0.93	0.95	0.96	0.97	0.96	0.95	0.93	**0.95**
	**RH**	0.94	0.97	0.94	0.97	0.93	0.96	0.97	0.94	0.94	0.96	0.97	0.97	0.95	0.93	**0.95**
	**FT**	0.95	0.96	0.95	0.96	0.95	0.96	0.98	0.96	0.95	0.96	0.98	0.97	0.96	0.94	**0.96**
	**RT**	0.95	0.96	0.96	0.94	0.95	0.96	0.96	0.94	0.96	0.93	0.96	0.96	0.93	0.95	**0.95**
	**Avg**	0.94	0.97	0.95	0.96	0.94	0.96	0.97	0.94	0.95	0.95	0.97	0.97	0.95	0.94	**0.95**
**Recall**	**LH**	0.94	0.95	0.94	0.95	0.93	0.96	0.96	0.95	0.94	0.93	0.97	0.96	0.93	0.93	**0.95**
	**RH**	0.94	0.96	0.94	0.96	0.94	0.95	0.97	0.94	0.95	0.96	0.97	0.97	0.94	0.93	**0.95**
	**FT**	0.95	0.97	0.95	0.97	0.94	0.97	0.98	0.94	0.95	0.96	0.98	0.97	0.97	0.94	**0.96**
	**RT**	0.94	0.97	0.95	0.95	0.95	0.97	0.96	0.94	0.96	0.96	0.96	0.96	0.95	0.95	**0.96**
	**Avg**	0.94	0.96	0.95	0.96	0.94	0.96	0.97	0.94	0.95	0.95	0.97	0.97	0.95	0.94	**0.95**
**F1-score**	**LH**	0.94	0.96	0.93	0.95	0.93	0.96	0.96	0.94	0.94	0.94	0.97	0.96	0.94	0.93	**0.95**
	**RH**	0.94	0.96	0.94	0.96	0.93	0.95	0.97	0.94	0.94	0.96	0.97	0.97	0.94	0.93	**0.95**
	**FT**	0.95	0.97	0.95	0.96	0.94	0.96	0.98	0.95	0.95	0.96	0.98	0.97	0.96	0.94	**0.96**
	**RT**	0.94	0.96	0.95	0.94	0.95	0.96	0.96	0.94	0.96	0.94	0.96	0.96	0.94	0.95	**0.95**
	**Avg**	0.94	0.96	0.94	0.95	0.94	0.96	0.97	0.94	0.95	0.95	0.97	0.97	0.95	0.94	**0.95**
**Misclassification Rate**		0.058	0.036	0.056	0.044	0.06	0.038	0.033	0.058	0.05	0.048	0.031	0.035	0.054	0.063	**0.0476**

**Table 3:** Accuracy comparison of classification performance on HGD dataset.

Methods/Subjects	1	2	3	4	5	6	7	8	9	10	11	12	13	14	Avg.
**DeepConvNet**	81.88	91.88	93.13	92.50	90.63	93.13	84.28	90.80	96.88	85.00	88.13	91.25	89.94	83.75	**89.51**
**EEGNet**	94.37	92.50	100	96.25	96.87	98.12	93.07	96.87	98.12	91.25	80.00	96.25	95.60	79.37	**93.47**
**CP-MixedNet**	88.75	90.00	95.63	91.25	95.00	91.25	88.05	93.13	95.00	88.75	75.63	93.75	89.31	78.13	**93.70**
**TS-SEFFNet**	90.69	93.53	98.53	96.88	92.90	93.53	92.40	91.78	96.88	89.88	92.78	95.40	93.03	87.34	**93.25**
**MBEEGNet**	95.02	95.02	100	99.40	98.17	98.80	93.13	95.52	98.18	92.14	89.43	96.02	94.45	88.88	**95.30**
**MBShallowCovNet**	98.25	96.23	98.80	98.18	97.65	96.90	93.80	97.00	97.52	92.50	80.78	96.25	95.62	92.04	**95.11**
**ANN + A-SVM**	94.2	96.4	94.4	95.6	93.9	96.2	96.7	94.2	94.9	95.2	96.9	96.5	94.6	93.7	**95.24**

**Table 4:** The comparison summary of classification performance among different models under different datasets.

Dataset	Methods		Accuracy (%)	F1-Score	Reference
	Feature Extraction	Classification			
**HGD**	ShallowConvNet	CNN	88.69	0.887	Schirrmeister, et.al [27] (2017)
	DeepConvNet	CNN	89.51	0.893	Schirrmeister, et.al [27] (2017)
	EEGNet	CNN	93.47	0.935	Lawhern, et al. [28] (2018)
	CP-MixedNet	CNN	93.70	0.937	Li, et.al [29] (2019)
	TS-SEFFNet	CNN	93.25	0.901	Li, et.al [29] (2019)
	MBEEGNet	CNN	95.30	0.954	Altuwaijri and Muhammad [30] (2022)
	MBShallowCovNet	CNN	95.11	0.951	Altuwaijri and Muhammad [30,31,32,33] (2022)
	**HOL–SSA–ORICA + CSP**	**ANN + A-SVM**	**95.24**	**0.95**	**-**

## Data Availability

Not applicable.
